# Tyrosine Phosphorylation of the BRI1 Receptor Kinase Occurs via a Post-Translational Modification and is Activated by the Juxtamembrane Domain

**DOI:** 10.3389/fpls.2012.00175

**Published:** 2012-08-08

**Authors:** Man-Ho Oh, Steven D. Clouse, Steven C. Huber

**Affiliations:** ^1^United States Department of Agriculture, Agricultural Research Service, University of IllinoisUrbana, IL, USA; ^2^Department of Plant Biology, University of IllinoisUrbana, IL, USA; ^3^Department of Horticultural Science, North Carolina State UniversityRaleigh, NC, USA

**Keywords:** autophosphorylation, hierarchical phosphorylation, brassinosteroid signaling, signal transduction, juxtamembrane domain

## Abstract

In metazoans, receptor kinases control many essential processes related to growth and development and response to the environment. The receptor kinases in plants and animals are structurally similar but evolutionarily distinct and thus while most animal receptor kinases are tyrosine kinases the plant receptor kinases are classified as serine/threonine kinases. One of the best studied plant receptor kinases is Brassinosteroid Insensitive 1 (BRI1), which functions in brassinosteroid signaling. Consistent with its classification, BRI1 was shown in early studies to autophosphorylate *in vitro* exclusively on serine and threonine residues and subsequently numerous specific phosphoserine and phosphothreonine sites were identified. However, several sites of tyrosine autophosphorylation have recently been identified establishing that BRI1 is a dual-specificity kinase. This raises the paradox that BRI1 contains phosphotyrosine but was only observed to autophosphorylate on serine and threonine sites. In the present study, we demonstrate that autophosphorylation on threonine and tyrosine (and presumably serine) residues is a post-translational modification, ruling out a co-translational mechanism that could explain the paradox. Moreover, we show that in general, autophosphorylation of the recombinant protein appears to be hierarchical and proceeds in the order: phosphoserine > phosphothreonine > phosphotyrosine. This may explain why tyrosine autophosphorylation was not observed in some studies. Finally, we also show that the juxtamembrane domain of BRI1 is an activator of the kinase domain, and that kinase specificity (serine/threonine versus tyrosine) can be affected by residues *outside* of the kinase domain. This may have implications for identification of signature motifs that distinguish serine/threonine kinases from dual-specificity kinases.

## Introduction

*Arabidopsis* contains more than 600 receptor-like kinases that are structurally and functionally similar to animal receptor kinases but are evolutionarily distinct. Animal receptor kinases are predominantly tyrosine kinases while plant receptor kinases are generally classified as serine/threonine kinases. However, recent work suggests that at least some plant receptor kinases are dual-specificity kinases that can autophosphorylate on serine, threonine, and tyrosine residues (Karlova et al., 2009; Oh et al., 2009, 2010). One of the most thoroughly studied plant receptor kinases is Brassinosteroid Insensitive 1 (BRI1), which functions with its co-receptor, BRI1-associated receptor kinase 1 (BAK1), in brassinosteroid (BR) signaling (Vert et al., 2005; Gendron and Wang, 2007; Clouse, 2011). BRI1 and BRI1-associated receptor kinase 1 (Oh et al., 2010) were the first two receptor kinases where tyrosine phosphorylation was shown to occur and to be of functional significance *in vivo*. Current thinking is that BRI1 and BAK1 are inactive and not interacting in the absence of BR, whereas in the presence of the ligand, BRI1 and BAK1 heterodimerize and become activated via auto- and transphosphorylation. The BRI1 kinase inhibitor 1 (BKI1; Jaillais et al., 2011), the BR-signaling kinase 1 (BSK1; Tang et al., 2008), and the constitutive differential growth 1 (CDG1) kinase (Kim et al., 2011) are thought to be the immediate downstream components that are first phosphorylated by BRI1. Phosphorylation of BKI1 releases the inhibitor protein, which enhances the ability of BRI1 to phosphorylate the receptor-like cytoplasmic kinases (RLCKs), BSK1, and CDG1. Both phosphorylated RLCKs can interact with the BRI1 suppressor 1 (BSU1) phosphatase (Mora-Garcia et al., 2004) and play a role in its activation, but CDG1 is apparently the protein kinase that phosphorylates BSU1 (Kim et al., 2011). BSK1 is thought to be an inactive kinase and may function as a scaffold to facilitate protein:protein interactions that activate BSU1, which then inhibits the glycogen synthase 3-like protein kinase, brassinosteroid insensitive 2 (BIN2), by dephosphorylation of an essential phosphotyrosine residue (Kim et al., 2009). The net result is that the transcription factors Brassinazole-Resistant 1 (BZR1; Wang et al., 2002; He et al., 2005) and BRI1-EMS suppressor 1 (BES1; Yin et al., 2002, 2005), also known as BZR2 (Wang et al., 2002) are dephosphorylated and able to move into the nucleus to up- or down-regulate the many genes that are BR-regulated (Vert et al., 2005; Nemhauser et al., 2006).

Our interest has been focused on the initial events in BR signaling, and in particular on the autophosphorylation of receptor kinases such as BRI1. As part of these studies we have further examined the mechanism of BRI1 autophosphorylation in order to understand two aspects in particular: (1) the activating role of the juxtamembrane domain; and (2) the paradox of tyrosine autophosphorylation. With respect to the juxtamembrane domain, we showed in a previous study (Oh et al., 2009) that the BRI1 kinase domain, in the absence of the juxtamembrane domain, could autophosphorylate on serine residues but not on threonine or tyrosine residues. This was interesting because many of the sites of threonine and tyrosine autophosphorylation reside within the kinase domain, but yet are not autophosphorylated if the entire juxtamembrane domain is removed. In the present study, we produced a nested series of juxtamembrane domain truncations to determine which sequences were essential for the activation. With respect to our second point of interest, the paradox of tyrosine autophosphorylation emerged when BRI1 was shown to contain phosphotyrosine (Oh et al., 2009) but autophosphorylation on tyrosine *in vitro* had not been observed to occur (Friedrichssen et al., 2000; Oh et al., 2000; Wang et al., 2005a). One plausible explanation to reconcile these conflicting observations would be that autophosphorylation on tyrosine is a co-translational, not post-translational, modification. Such a mechanism has, in fact, been demonstrated for glycogen synthase kinase 3 (GSK3; Cole et al., 2004; Lochhead et al., 2006) and dual-specificity tyrosine-phosphorylation-regulated protein kinase (DRKY; Lochhead et al., 2005). The GSK3 and DRKY family protein kinases autophosphorylate on tyrosine residues as transitional intermediates while the mature proteins can only catalyze phosphorylation on serine/threonine residues and thus contain phosphotyrosine but only phosphorylate serine/threonine residues. We explored this possibility with the recombinant BRI1 cytoplasmic domain (CD) and show in the present report that BRI1 autophosphorylation on serine, threonine, and tyrosine residues occurs as a result of a post-translational, rather than co-translational, modification. One line of evidence to support this conclusion comes from the observation that *E. coli* proteins are phosphorylated on serine, threonine, and tyrosine residues during production of recombinant BRI1. This was reported recently (Oh et al., 2012a) and in the present study we further characterize the time course of transphosphorylation of *E. coli* proteins during production of recombinant BRI1. The potential for BRI1 to catalyze transphosphorylation of proteins on tyrosine residues (in addition to serine and threonine residues) emerges as an additional component that may contribute to BR signaling in planta and supports the recent report of BRI1-mediated phosphorylation of BKI1 at Tyr-211 (Jaillais et al., 2011).

## Materials and Methods

### Expression of recombinant receptor kinase cytoplasmic domains in *E. coli*

The CDs of HAESA (At4g28490; residues 645–999), SRF9 (At1g11130; residues 365–768), FLS2 (At5g46330; residues 830–1173), At2g23950 (residues 259–634), BRI1 (At4g39400; residues 815–1196), BAK1 (At4g33430; residues 249–615), BKK1 (At2g13790; residues 255–620), BRL1 (At1g55610; residues 800–1166), or BRL3 (At3g13380; residues 797–1164) were cloned into a Gateway modified pFlag-Mac vector and expressed in BL21 (DE3) pLysS cells (Novagen, Rockland, MA, USA). As specified, serial truncations of the juxtamembrane domain of BRI1 CD were made by sequentially removing five amino acid segments between residue 815 and 875. The truncation mutants were cloned and expressed as for the intact CDs. The recombinant Flag-tagged proteins were immunopurified using anti-Flag M2 affinity gel (Sigma-Aldrich, St. Louis, MO, USA). After elution from the beads, the protein solutions were dialyzed against a 1000× volume of buffer containing 20 mM MOPS, pH 7.5, and 1 mM DTT as previously described (Oh et al., 2000).

### Electrophoresis and immunoblotting

Recombinant protein preparations were mixed with four-volumes of pre-heated (95°C) 1× SDS-PAGE sample buffer containing 1 M urea, 0.7 M 2-mercaptoethanol, 5 mM NaF, 1 mM Na_2_MoO_4_, 1 mM Na_3_VO_4_, 1 mM aminoethylbenzenesulfonyl fluoride, and 2 mM EDTA. Protein concentrations were determined by the dye-binding assay (Bio-Rad, Hercules, CA, USA) with bovine serum albumin as the standard. Proteins were separated on 12% polyacrylamide (0.1% SDS) gels and transferred to polyvinylidene difluoride (PVDF) fluorescence-specific membranes (Millipore, Bedford, MA, USA). Membranes were blocked in a 2% (v/v) fish gelatin solution in phosphate-buffered saline (PBS; 5 mM NaH_2_PO_4_, 150 mM NaCl, pH 7.4) before being incubated with primary antibodies, which were diluted as specified in PBS containing 0.1% (v/v) Tween-20 (PBST). Blots were probed with custom polyclonal antibodies that recognize BRI1 phosphotyrosine-831 (pY831; 1:1000 dilution), phosphotyrosine-956 (pY956; 1:1000 dilution), or phosphotyrosine-1072 (pY1072; 1:1,000 dilution; Oh et al., 2009), or phosphoserine-858 (pS858; 1:2,000 dilution), phosphothreonine-872 (pT872; 1:3,000 dilution), or phosphoserine-891 (pS891; 1:2,000 dilution; Oh et al., 2012b). Alternatively, blots were stained with ProQ Diamond phosphoprotein stain (Invitrogen, Grand Island, NY, USA) or Coomassie Brilliant Blue (CBB) as specified. After incubation with primary antibodies for 1 h at room temperature, membranes were washed with PBST and incubated with an Alexa Fluor 680- or IR 800-conjugated secondary antibody (Rockland Immunochemicals, Gilbertsville, PA, USA) diluted at 1:20,000 in PBST. Immunoblot imaging and densitometry analysis was performed using a LI-COR Odyssey^®^ Infrared Imaging System (LI-COR Biosciences, Lincoln, NE, USA). In general, experiments were performed at least twice and representative results are presented.

### 2-Dimensional electrophoresis

*E. coli* cells expressing Flag-BRI1 CD were harvested before addition of IPTG or 16 h after addition of IPTG to induce recombinant protein production. Bacterial cells were lysed and debris was removed by low speed centrifugation. The majority of the Flag-BRI1 protein was removed by absorption using anti-Flag M2 affinity gel (Sigma-Aldrich, St. Louis, MO, USA) and the remaining proteins were processed for 2-DE. Total *E. coli* proteins were precipitated with five volumes of ice-cold 0.1 M ammonium acetate in methanol and left at −20°C overnight to precipitate total soluble proteins. After centrifugation at 24,000 *g* for 20 min, the protein pellet was washed three times with 1 ml with ice-cold 0.1 M ammonium acetate in methanol, and once with ethanol before resuspension in 300 μl IEF sample loading buffer (30 mM Tris-HCl, pH 8.5; 7 M urea, 2 M thiourea, 4% CHAPS) with vortexing. The resuspending pellet was then incubated at 30°C for 1 h, centrifuged at 24,000 g for 20 min, and the resulting supernatant was transferred to a new tube. The protein concentration was determined with Bio-Rad protein assay using BSA as a standard and the 2-DE analysis was performed as described (Oh et al., 2009). Total protein (300 μg) was loaded onto Immobiline™ DryStrip pH 3–10 (13 cm) strips and focused on the IPGphor IEF system for a total of 20 h (68 kVh) at 20°C. After focusing, the strips were equilibrated for 10 min in 10 ml of equilibration buffer containing first DTT followed by buffer containing iodoacetamide. The strips were then placed onto 12% (w/v) SDS-PAGE gels and run for 6 h.

### Peptide kinase activity

Peptide substrate phosphorylation assays were performed as described (Oh et al., 2000) using 80 μg ml^−1^ SP11 peptide as substrate (sequence: GRJRRIASVEJJK, where J is norleucine; produced by Bethyl Laboratories, Montgomery, TX, USA). Reactions were incubated at room temperature and peptide kinase activity was measured using the filter paper binding assay. Values reported are means ± SEM and are representative of at least two independent experiments.

### Mass spectrometry

Proteins in 2-DE gel slices were digested with trypsin (Promega, Madison, WI, USA) for 16 h and the tryptic peptides were extracted with 0.5% TFA and 50% acetonitrile and dried. The collected peptides were further purified with C18 Ziptips (Millipore, Billerica, MA, USA), before analysis in the Voyager DE-STR MALDI-TOF (Applied Biosystems, Carlsbad, CA, USA). The matrix was 1% phosphoric acid in 2,5-dihydroxybenzoic acid (DHB; Kjellström and Jensen, 2004) to promote phosphopeptides detection, and spectra were collected at the mass window 600–3000 m/z in the linear positive mode. Peaks were smoothed with the Gaussian method, and the mass fingerprint of each spot was searched in ProFound (Zhang and Chait, 2000). The NCBI nr database with a taxonomy limited to *E. coli* proteins was used. Mass tolerance was set at 1.0 Da, with methionine oxidation as a variable modification. The protein hits with best *E*-values are reported, and all were further validated to insure that the theoretical pI and MW of the protein matched the position of the spot in the 2-DE gel.

## Results

### Autophosphorylation of BRI1 in *E. coli*

**Figure 1:**
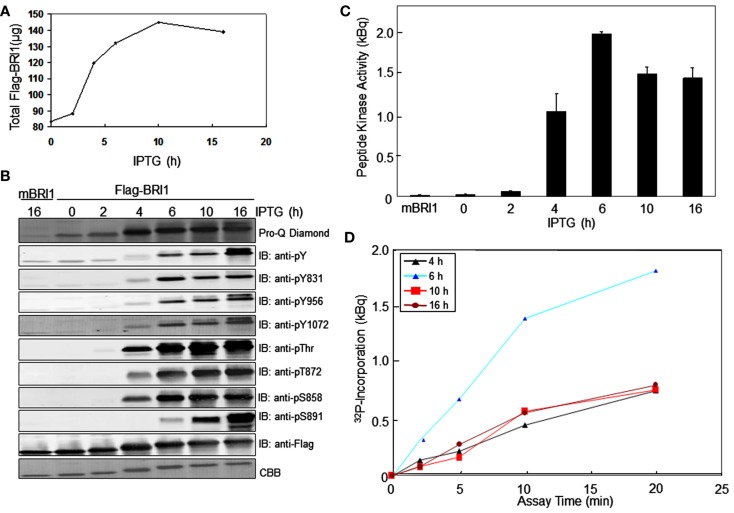
**Time course of Flag-BRI1 production in *E. coli***. **(A)** Total recovery of recombinant protein from bacterial cell cultured for up to 16 h after addition of (?) mM IPTG at 22°C. **(B)** Immunoblot analysis of purified Flag-BRI1 protein harvested from cells at different stages of induction, using a variety of generic and modification-specific antibodies. Staining with ProQ Diamond phosphoprotein stain reported phosphorylation at all serine, threonine, and tyrosine sites, and immunoblotting with anti-Flag antibodies and staining with Coomassie Brilliant Blue (CBB) demonstrated equal protein loading (1.5 μg per lane) and the electrophoretic mobility shift accompanying autophosphorylation. **(C)** Peptide kinase activity of BRI1 purified at different stages of induction. The SP11 peptide (sequence: GRJRRIASVEJJKK, where J is norleucine) was used and reaction mixtures were incubated for 20 min at room temperature. **(D)** Time courses of SP11 peptide phosphorylation catalyzed by BRI1-Flag purified at different stages of induction.

**Figure 2:**
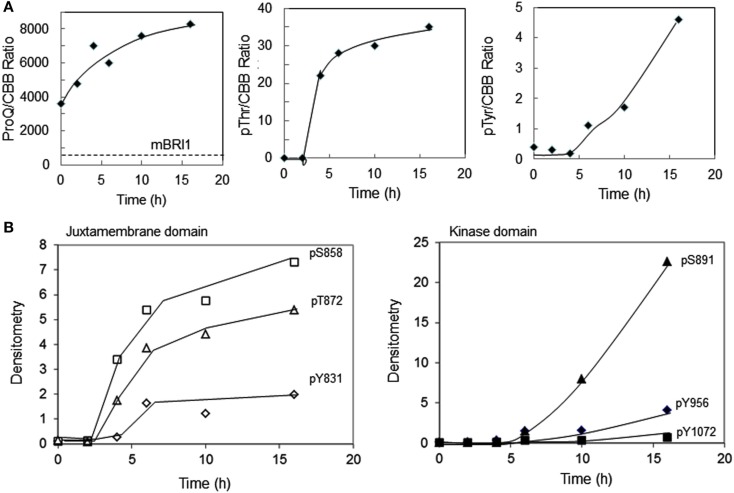
**Quantification of Flag-BRI1 autophosphorylation during induction in *E. coli***. **(A)** Densitometric analysis of immunoblot data in Figure 1 showing the increase in ProQ Diamond staining, and cross-reaction with anti-phosphothreonine or anti-phosphotyrosine antibodies, normalized for Flag-BRI1 protein based on detection by CBB staining. The dashed line in the left panel is the value obtained for the kinase-inactive BRI1 (K911E) directed mutant, which is completely unphosphorylated but reacts weakly with ProQ Diamond stain and provides a baseline for interpretation of the autophosphorylation of active Flag-BRI1. **(B)** Densitometry analysis of phosphorylation of specific sites based on immunoblotting with custom antibodies as in Figure 1. Residues monitored that are contained within the JM domain and KD are plotted separately as indicated.

We monitored the autophosphorylation status of Flag-BRI1as a function of time during production of the recombinant protein in *E. coli* using generic and custom phospho-specific antibodies. There was a significant amount of Flag-BRI1 protein present in uninduced cells and following the addition of IPTG there was an accumulation of recombinant protein until 10 h (Figure 1A). The Flag-BRI1 protein isolated from uninduced cells (time zero) was relatively unphosphorylated as indicated by weak staining with ProQ Diamond phosphoprotein stain and essentially no cross-reactivity with anti-phosphotyrosine or phosphothreonine antibodies (Figure 1B). However, when normalized for protein, the Flag-BRI1 protein from uninduced *E. coli* cells had fivefold higher staining with ProQ Diamond than did the kinase-inactive Flag-BRI1 (K911E) directed mutant, referred to as mBRI1, that is completely unphosphorylated (Wang et al., 2005a). These results suggested that the BRI1 protein present in uninduced cultures was phosphorylated to only a limited extent and primarily on serine residues. The stoichiometry of autophosphorylation of Flag-BRI1 increased progressively during IPTG-induction as evidenced by: (i) increased ProQ Diamond staining and (ii) reduced migration during SDS-PAGE. At time zero, the electrophoretic mobility of Flag-BRI1 was only slightly reduced relative to that of mBRI1 (Figure 1B), consistent with the notion that the protein was already partially phosphorylated at time zero. Reduced migration of the protein was clearly apparent after 2 h of induction, but substantial cross-reactivity with anti-phosphothreonine antibodies was first observed at 4 h, and anti-phosphotyrosine antibodies at 6 h (Figure 1B). Collectively, these results suggest that in general, autophosphorylation occurs first on serine, followed by threonine, and then tyrosine residues (Figure 2A). However, the kinetics of autophosphorylation on specific serine and tyrosine residues differed significantly from these general overall patterns. For example, while autophosphorylation on serine residues was present at time zero and increased immediately after IPTG addition, autophosphorylation at the Ser-858 site was first evident at 4 h and autophosphorylation at Ser-891 was first observed at 6 h (Figures 1B and 2B). Likewise, phosphorylation at the Tyr-831 site plateaued at 6 h whereas phosphorylation at Tyr-956 and -1072 continued to increase with time of induction. Of the specific sites monitored, autophosphorylation of residues in the JM domain paralleled one another (Figure 2B left panel) and preceded autophosphorylation of the specific sites monitored in the kinase domain (Figure 2B right panel), which occurred later and continued to increase with time.

We also monitored the peptide kinase activity of Flag-BRI1 purified at different times of IPTG-induction (Figure 1C). Significant transphosphorylation activity of Flag-BRI1 with the SP11 synthetic peptide as substrate (Oh et al., 2000) was first observed at 4 h of induction and maximum activity was observed in the 6-h sample; after longer induction periods the peptide kinase activity was reduced. The increase in peptide kinase activity up to 6 h presumably reflects autophosphorylation of sites essential for kinase activity and likely involves the serine/threonine residues in the activation loop (Oh et al., 2000; Wang et al., 2005a,b) that are required for activity of this RD-type kinase. We found that the partially autophosphorylated protein purified at time zero or after 2 h of induction did not autophosphorylate further and activate during the peptide kinase assay, as activity remained low during a 20-min assay. Likewise, the partially activated enzyme purified after 4 h of induction had linear rates of peptide phosphorylation *in vitro* (Figure 1D) indicating that essential sites were not autophosphorylated *in vitro*. The decrease in activity at 10 and 16 h of induction presumably involves autophosphorylation of residue(s) that inhibit peptide kinase activity, and of the sites monitored, Ser-891 (Oh et al., 2012b) and Tyr-956 would be potential candidates.

### Transphosphorylation of *E. coli* proteins during expression of receptor kinases

**Figure 3:**
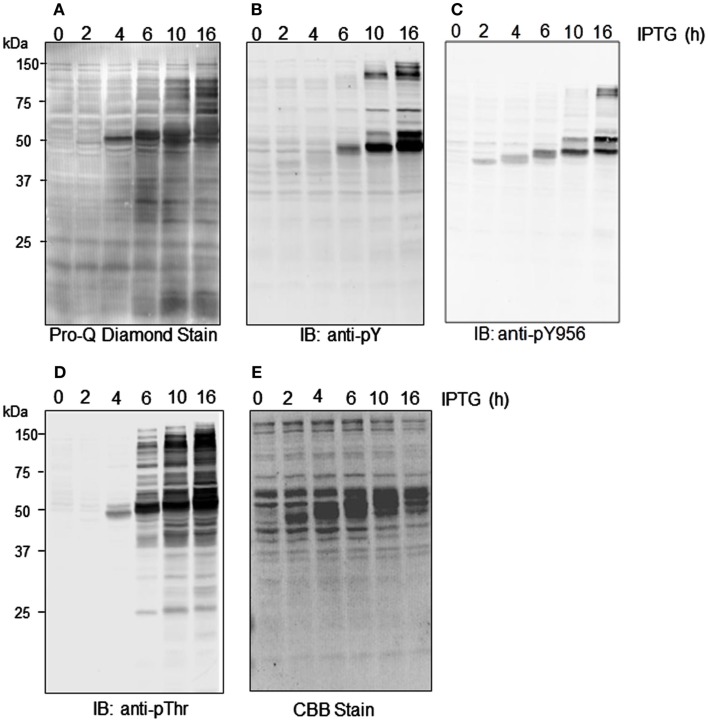
**Time course of transphosphorylation of *E. coli* proteins during expression of Flag-BRI1 cytoplasmic domain**. At each time point, Flag-BRI1 protein was immunopurified and the remaining supernatant (containing *E. coli* proteins and some residual Flag-BRI1 protein) was resolved by SDS-PAGE followed by staining or immunoblot analysis. **(A)** ProQ Diamond phosphoprotein stain. **(B)** Immunoblotting (IB) with anti-phosphotyrosine (anti-pY) or **(C)** anti-phosphotyrosine-956 (anti-pY956), or **(D**) anti-phosphothreonine (anti-pT) antibodies. **(E)** Staining with Coomassie Brilliant Blue (CBB).

We recently reported that *E. coli* proteins can be phosphorylated by Flag-BRI1 during induction of the recombinant protein (Oh et al., 2012a). This observation is confirmed here and further characterized as a function of time during the induction process. As shown in Figure 3, there was little phosphorylation of *E. coli* proteins at time zero based on weak staining with ProQ Diamond phosphoprotein stain and little or no cross-reaction of the bacterial proteins with generic phospho-specific antibodies. However, at 4 h of induction autophosphorylation of the Flag-BRI1 protein at ∼50 kDa became apparent and thereafter was the dominant phosphoprotein band on the blot. Moreover, with increasing induction time *E. coli* proteins were clearly phosphorylated as well as evidenced by ProQ Diamond staining (Figure 3A) and cross-reaction of numerous proteins (outside the ∼50-kDa range) with anti-phosphotyrosine (Figure 3B) and anti-phosphothreonine (Figure 3D) antibodies. Apparent transphosphorylation of *E. coli* proteins was evident at 6 h and increased with time of induction despite the fact that the contribution of *E. coli* proteins on the blot was decreasing as production of the recombinant protein increased with time (all lanes in Figure 3E were loaded with equal amounts of total protein). The array of *E. coli* proteins transphosphorylated on serine, threonine, and tyrosine was clearly different; the fewest proteins were phosphorylated on tyrosine and were all relatively large proteins while a greater number were phosphorylated on threonine residues and included proteins with a wider range of molecular masses (Figure 3D). ProQ Diamond stained a number of proteins that were not detected by the anti-phosphothreonine antibodies and presumably were phosphorylated on seryl residues, including for example a number of proteins well below 25 kDa. Custom antibodies specific for phosphorylation of BRI1 at the tyrosine-956 site cross-reacted with Flag-BRI1 protein, as expected (Oh et al., 2009), but only weakly cross-reacted with a few discrete proteins of higher molecular weight (∼75 kDa; Figure 3C), and these proteins were not detected with the generic anti-phosphotyrosine antibodies (Figure 3B). The likely explanation is that there was some phosphorylation of *E. coli* proteins at sites with sufficient sequence similarity to the BRI1 Tyr-956 site that the custom antibodies cross-reacted, but the level of phosphorylation was too low to result in appreciable cross-reaction with the generic anti-phosphotyrosine antibodies. Importantly, these results higher molecular weight proteins containing phosphotyrosine did not cross react with anti-Flag antibodies (data not shown) confirming that they were not cross-linked or dimeric forms of Flag-BRI1, but rather represent transphosphorylation of *E. coli* proteins catalyzed by Flag-BRI1.

**Figure 4:**
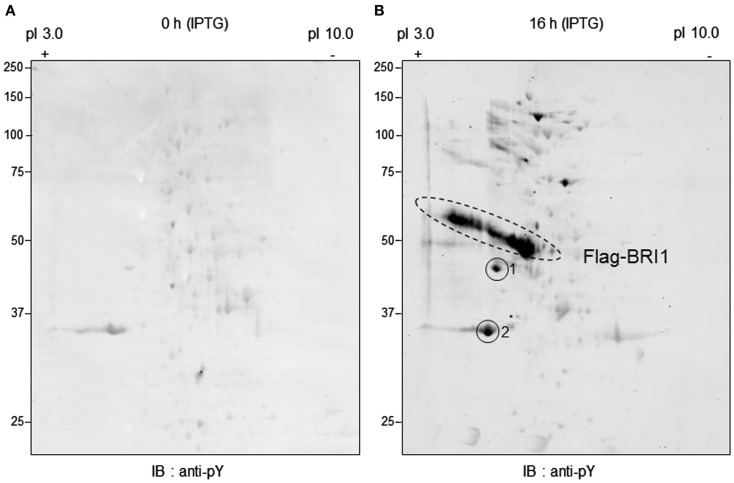
**Separation of *E. coli* proteins by 2-DE followed by immunoblot analysis with anti-phosphotyrosine antibodies**. Samples were collected at **(A)** 0 h and **(B)** 16 h following addition of IPTG to induce Flag-BRI1 production. From each sample, the majority of the Flag-BRI1 protein was removed by immunopurification, and the remaining supernatant (300 μg protein) was subjected to 2-DE analysis.

To further characterize the transphosphorylation of *E. coli* proteins by recombinant BRI1, total soluble proteins from bacterial cells at time zero, or after 16 h of induction were resolved by 2-DE and blots were probed with anti-phosphotyrosine antibodies (Figure 4). There was little cross-reaction with proteins before induction (Figure 4A) compared to strong cross-reaction with numerous discrete proteins 16 h post IPTG addition (Figure 4B), suggesting that Flag-BRI1 can transphosphorylate *E. coli* proteins on tyrosine residues *in situ*. Protein contained in spots 1 and 2 (Figure 4B) was digested with trypsin and analyzed by MALDI-ToF-MS (Table A1 in Appendix). Spot 1 was found to contain a soluble periplasmic maltose-binding protein, which functions in conjunction with a multi-subunit membrane transporter MalFGK_2_ (Cui et al., 2010). Spot 2 contained chain A of the OmpF protein, which is one of the two major outer membrane proteins in *E. coli* that functions as a homotrimer to form a transmembrane pore in the outer membrane providing passive diffusion pores (Cowan et al., 1992). Unfortunately, it has not been possible in attempts thus far to identify the site(s) of tyrosine phosphorylation for either Spot 1 or 2, whether phosphorylation of these proteins had any impact on their function. It is also worth noting that neither of the spots contained peptides that could have been derived from BRI1, which is consistent with the notion that tyrosine phosphorylation was occurring on *E. coli* proteins, and was not an artifact caused by truncated forms of BRI1 protein.

It is also interesting to note that the recombinant Flag-BRI1 protein resolved into a series of spots along a diagonal line (Figure 4B), consistent with the altered migration on one-dimensional electrophoresis associated with the highly autophosphorylated protein (e.g., Figure 2). The fact that Flag-BRI1 protein was found in a diagonal charge train while tyrosine-phosphorylated *E. coli* proteins were contained in discrete spots also indicates that the additional spots are not fragments or cross-linked forms of Flag-BRI1, as in that scenario one might expect to find partial charge trains at lower or higher molecular weights compared to authentic Flag-BRI1 and this was not observed.

**Figure 5:**
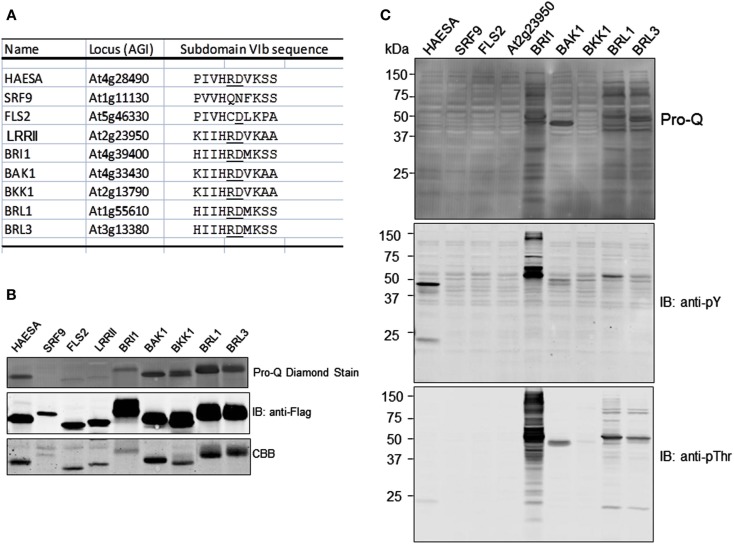
**Not all receptor kinases can transphosphorylate *E. coli* proteins during induction**. **(A)** Alignment of subdomain VIb for the seven RD-type and two non-RD-type (SRF9 and FLS2) receptor kinases used in the present study. The RD dipeptide motif, when present, is underlined. **(B)** Autophosphorylation analysis of the nine receptor kinases, all expressed as fusion proteins with an N-terminal Flag tag, as analyzed by staining with ProQ Diamond phosphoprotein stain. **(C)** Transphosphorylation of *E. coli* proteins after 16 h of expression of the indicated receptor kinase. For each sample, the Flag-tagged receptor kinase was imunopurified and the remaining supernatant was subjected to SDS-PAGE, transferred to PVDF, and stained with ProQ Diamond.

We also tested the CDs of several other leucine-rich repeat receptor-like kinases (LRR-RLKs) for their ability to transphosphorylate *E. coli* proteins. The group of receptor kinases tested included both RD and non-RD-type kinases (Figure 5A). The RD kinases are named for the Arg-Asp dipeptide motif in subdomain VIb, which is typically found in protein kinases that require autophosphorylation of residue(s) within the activation loop for the kinase to be active (Johnson et al., 1996). As shown in Figure 5B, six of the RD-type kinases (HAESA, BRI1, BAK1, BKK1, BRL1, and BRL3) were capable of autophosphorylation, while one was not (At2g23950; labeled as LRRII) and was similar to the non-RD kinases (SRF9 and FLS2) that also did not autophosphorylate during production in *E. coli* or after incubation with ATP *in vitro* (data not shown). The basis for the lack of autophosphorylation of the RD-type kinase, At2g23950, could indicate that the recombinant protein did not fold properly, that phosphorylation of activation loop residues is catalyzed by another protein kinase, or simply that autophosphorylation does not occur with this particular kinase. For the three proteins that did not autophosphorylate (by ProQ Diamond staining), immunoblotting with anti-Flag antibodies cross-reacted with single bands although in each case Coommassie staining detected minor contaminating proteins that were not related to the recombinant protein kinase.

Following purification of the recombinant CDs, the remaining soluble-protein supernatants (containing *E. coli* proteins and a variable amount of residual recombinant protein) were analyzed for protein phosphorylation to identify which receptor kinases could catalyze transphosphorylation of *E. coli* proteins. Of those tested, BRI1 could strongly transphosphorylate on threonine and tyrosine residues as expected, while BRL1 and BRL2 could only transphosphorylate *E. coli* proteins weakly on threonine residues and not at all on tyrosine. Because transphosphorylation of *E. coli* proteins by BRL1 and BRL3 onthreonine residues was markedly reduced relative to BRI1, but total transphosphorylation as monitored by ProQ Diamond staining was similar among the three receptor kinases, it would appear that BRL1 and BRL3 are primarily transphosphorylating *E. coli* proteins on serine residues. Thus, it appears that the three receptor kinases may differ in specificity of transphosphorylation (serine versus threonine versus tyrosine). It is also worth noting that inability of some receptor kinases (i.e., BAK1, BKK1, and HAESA) to transphosphorylate *E. coli* proteins cannot be attributed to inherent lack of catalytic activity, and therefore autophosphorylation and transphosphorylation activities are not directly related to one another.

### The juxtamembrane domain is an activator of the BRI1 kinase domain

**Figure 6:**
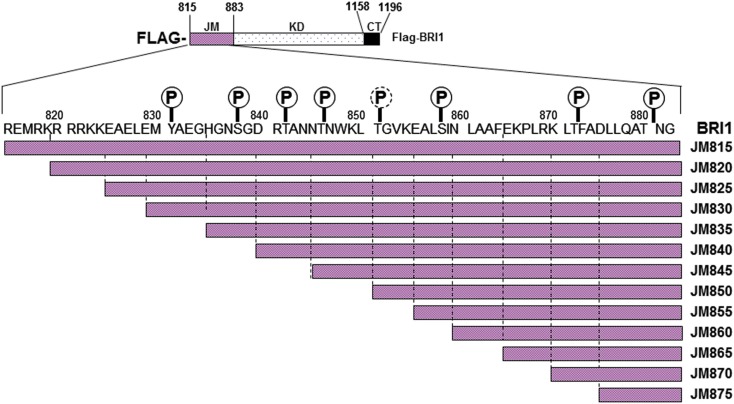
**Schematic representation of the primary structure of Flag-BRI1 cytoplasmic domain showing the serial truncations made through the JM domain**. The positions of confirmed and possible phosphorylation sites are indicated with solid or dashed symbols, respectively.

**Figure 7:**
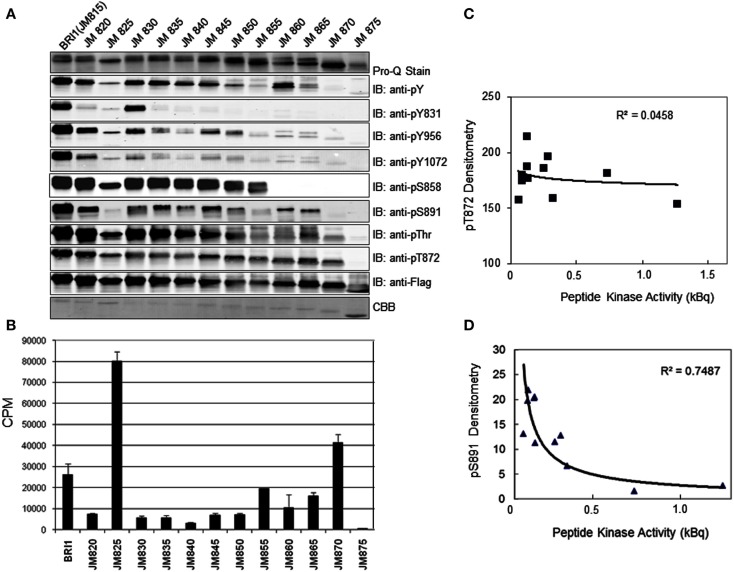
**The JM domain is an activator of the BRI1 kinase domain**. Serial deletions of the JM domain were constructed to produce truncation mutants starting with the indicated residue. **(A)** Analysis of autophosphorylation of the JM truncation mutants by staining with ProQ Diamond and immunoblotting with generic and custom antibodies. **(B)** Peptide kinase activity of the JM truncation mutants measured using the SP11 synthetic peptide. Values are means of three determinations ± SEM. **(C)** Correlation between peptide kinase activity (from B) and relative phosphorylation of the threonine-872 site, determined by densitometric analysis of the immunoblots in A. **(D)** Correlation between peptide kinase activity and relative phosphorylation status of the serine-891 site.

Previous studies identified the juxtamembrane domain as an activator of both the auto- and transphosphorylation activity of BRI1 (Oh et al., 2009). As an initial approach to determine which regions of the juxtamembrane domain are crucial to the activation of kinase activity, we constructed a nested series of truncations that sequentially removed five amino acid segments from the N-terminus of this flanking region (Figure 6). The full-length Flag-BRI1 CD (with a complete juxtamembrane domain) starts at residue 815 and was strongly autophosphorylated as indicated by robust staining with ProQ Diamond and cross-reaction with all of the general and specific antibodies used (Figure 7A). Removal of the majority of the juxtamembrane domain (as in the JM875 truncation, which retains only the last eight residues of the juxtamembrane domain) produced a protein that was stained with ProQ Diamond but did not cross react with any of the general or specific antibodies directed against phosphotyrosine or phosphothreonine residues, which is similar to the result obtained by complete removal of the juxtamembrane domain (Oh et al., 2009). The ProQ Diamond staining of the JM875 truncation mutant presumably reflects autophosphorylation on serine residues, but did not include Ser-858 and -891, which were monitored specifically. Autophosphorylation on threonine residues became apparent when the juxtamembrane domain was extended to residue 870 but the first evidence for tyrosine phosphorylation required the juxtamembrane domain to be extended slight further (to residue 865). Further extension of the domain did not alter the level of autophosphorylation on threonine residues (including the Thr-872 site) while tyrosine phosphorylation varied substantially. As expected, autophosphorylation of residues located in the juxtamembrane domain (Tyr-831, Thr-872, and Ser-858) was only observed in constructs that included the corresponding residue providing additional evidence for the specificity of the modification-specific antibodies. In the case of Tyr-831, the construct with only one adjacent N-terminal residue was sufficient for maximum autophosphorylation at this site (the JM830 truncation in Figure 7A); when the juxtamembrane domain was extended 5 or 10 residues further, phosphorylation on Tyr-831 was strongly attenuated and only recovered when the final five residues of the juxtamembrane domain were restored. Clearly, residues within the general vicinity of sites of tyrosine autophosphorylation can strongly influence their autophosphorylation activity, and it is interesting that sites of threonine phosphorylation were overall much less affected than sites of tyrosine autophosphorylation when length of the juxtamembrane domain was varied.

We also monitored peptide kinase activity of the various juxtamembrane domain truncation mutants and found remarkable variation in the activities observed (Figure 7B). Consistent with our earlier findings (Oh et al., 2009), the shortest construct (JM875 truncation) had essentially no peptide kinase activity relative to the full-length Flag-BRI1 CD that readily transphosphorylated the peptide substrate. Of the other truncations, only a relatively short JM (as in the JM870 truncation) or substantially longer JM (as in the JM825 truncation) had high rates of peptide kinase activity. It is worth noting that truncation of the JM domain affected transphosphorylation of the peptide substrate (Figure 7B) and overall autophosphorylation (ProQ Diamond staining, Figure 7B) differently as truncations JM830 through JM850 had strong autophosphorylation but very low transphosphorylation (i.e., peptide kinase) activity. It was visually apparent that the variation in peptide kinase activity was inversely related to the intensity of autophosphorylation at the serine-891 site. Indeed, when we quantified the immunoblot signals for each JM truncation (Figure 7A) and compared with the peptide kinase activity of that construct, the strongest correlation was with the phosphorylation status of serine-891 (negative correlation, *R*^2^ = 0. 7487, Figure 7D), which is consistent with the notion that phosphorylation of serine-891 inhibits kinase domain activity (Oh et al., 2012b). There was no correlation between peptide kinase activity and phosphorylation of the threonine-872 site (*R*^2^ = 0.0458, Figure 7C).

**Figure 8:**
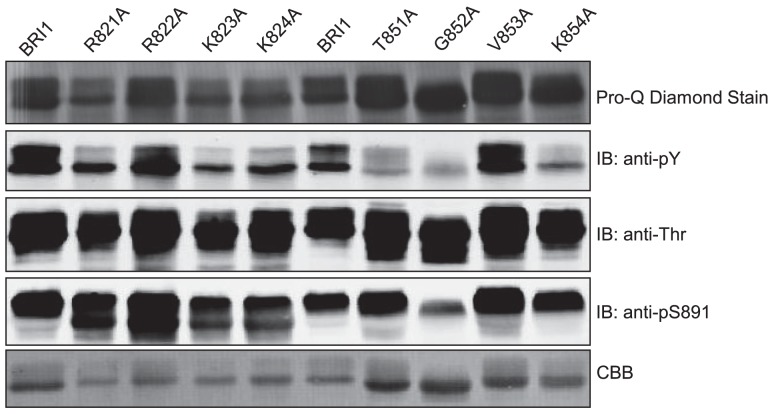
**Identification of specific JM domain residues that are essential for autophosphorylation of BRI1 on tyrosine residues**. Two critical regions identified by truncation analysis were examined by alanine scanning of individual residues in the full-length Flag-BRI1 cytoplasmic domain. The recombinant proteins were expressed and autophosphorylation was analyzed by ProQ Diamond staining and immunoblotting with generic phosphotyrosine and phosphothreonine antibodies.

Based on the analysis of truncation mutants, we selected two regions of the JM domain where adjacent truncations had a particularly marked difference in tyrosine autophosphorylation and did alanine scanning through the critical region to identify specific residues within the segment that are essential for overall tyrosine autophosphorylation. For example, comparing the JM820 and JM825 truncation mutants suggests that residues between 820 and 825 are essential for tyrosine phosphorylation (Figure 7A), and indeed, substitution of Arg-821, Lys-823, or Lys-824 with alanine dramatically reduced tyrosine autophosphorylation with much less reduction in threonine phosphorylation or ProQ Diamond staining (Figure 8), establishing that specific residues within this region affect tyrosine autophosphorylation even with the full-length Flag-BRI1 CD. Likewise, we did alanine scanning through the region bracketed by the truncation mutants JM850 and JM855 (Figure 7A) and established that Thr-851, Gly-852, and Lys-854 are also virtually essential for autophorylation on tyrosine, but not threonine, residues (Figure 8). Both regions had residues (e.g., arginine-822 and valine-853) that when substituted with alanine had little effect on autophosphorylation indicating that not all residues were essential. The dramatic effect of substituting alanine for lysine or arginine at certain positions suggests that positive charges may play a role but the large effect of the conservative substitutions (e.g., T851A and G852A) is surprising. In the case of Thr-851, substitution with alanine would prevent autophosphorylation at this site, and indeed this residue was identified as a possible *in vivo* phosphorylation site (Wang et al., 2005a). However, the dramatic effect of substitution of alanine for Gly 852 would not be predicted but indicates that subtle side chain differences or propensity to adopt secondary structure may be important factors.

## Discussion

The results of the present study provide new insights to the process of activation of the BRI1 receptor kinase by autophosphorylation. Specifically, we demonstrate that BRI1autophosphorylation: (i) is hierarchical; (ii) is post-translational rather than co-translational; (iii) likely involves both intra- and inter-molecular events; and (iv) is activated by the juxtamembrane domain. The hierarchical nature of autophosphorylation was evident during production of recombinant Flag-BRI1 in *E. coli*, where two generalizations emerged. First, overall autophosphorylation proceeded in the temporal order: serine > threonine > tyrosine (Figure 2A), although autophosphorylation of several specific phosphosites did not follow this general pattern. Second, autophosphorylation of residues within the juxtamembrane domain preceded residues in the kinase domain (Figure 2B). Exceptions to the latter generalization would be residues located within the activation loop, which are required for activity and therefore must be phosphorylated prior to any other sites. However, we were monitoring specific sites using custom antibodies and thus were unable to specifically monitor the phosphorylation of activation loop residues. If autophosphorylation was not hierarchical, one would observe all autophosphorylation sites increasing in stoichiometry concurrently, and this was clearly not observed (Figure 1B). It will be interesting to see whether autophosphorylation *in vivo* also appears to be hierarchical. However, the results obtained from *in vivo* experiments may be confounded by the fact that BRI1 is a rather long-lived protein that is simultaneously subject to both autophosphorylation and dephosphorylation potentially by a variety of protein phosphatases, including protein phosphatase 2A (Wu et al., 2011). Thus, it may be difficult to monitor the initial autophosphorylation reactions following synthesis *in vivo*, but the studies reported here with recombinant protein provide a firm foundation for future studies in planta.

The question of post- versus co-translational autophosphorylation was of most concern with respect to modification of tyrosine residues and was the essence of the present study. As noted in the Introduction, recombinant BRI1 was observed to contain phosphotyrosine but in studies of *in vitro* autophosphorylation, only ^32^P-labeling of serine and threonine residues was observed, and thus emerged the paradox of tyrosine autophosphorylation. One resolution to the paradox would be that autophosphorylation on tyrosine residues at least was a co-translational process, as observed for the GSK3 and DRKY family protein kinases. Several lines of evidence suggest that autophosphorylation of Flag-BRI1 protein on serine, threonine, and tyrosine residues occurs as a result of a traditional post-translational modification. First, if autophosphorylation was co-translational there would be no increase in autophosphorylation during induction; i.e., autophosphorylation normalized for BRI1 protein would be constant and that is not the case (Figures 1B and 2A). Second, uninduced bacterial cells contained a substantial amount of Flag-BRI1 protein that was partially phosphorylated but only on serine residues and that protein disappeared during induction presumably via autophosphorylation and conversion to the more slowly migrating form(s) on SDS-PAGE (Figure 1B). Third, the mature Flag-BRI1 protein can transphosphorylate *E. coli* proteins on serine, threonine, and tyrosine residues (Figures 4 and 5C) and this is not consistent with the co-translational mechanism. Transphosphorylation of *E. coli* proteins *in situ* generally tracks the ability of the purified Flag-BRI1 protein to transphosphorylate the SP11 peptide *in vitro* (compare Figures 1C and 3). The ability of BRI1 to transphosphorylate certain proteins on serine and threonine residues was recognized to occur (Nam and Li, 2004; Ehsan et al., 2005) but transphosphorylation on tyrosine lends strong support to the post-translational mechanism. As our studies were underway, it was reported that one of the earliest steps in BR signaling involves BRI1 phosphorylation of the BKI1 protein on Tyr-211, which is located in the membrane-binding domain of the inhibitor protein, causing release from the plasma membrane (Jaillais et al., 2011). Thus, the BRI1-mediated transphosphorylation of downstream components on tyrosine residues is established. It is possible that other proteins are also transphosphorylated by BRI1 on tyrosine residues *in vivo* and will be the subject of future experiments.

The mechanism of autophosphorylation can be either intra- or inter-molecular in nature, and both have been suggested as mechanisms for recombinant BRI1 autophosphorylation *in vitro*. For example, working with BRI1 expressed as an N-terminal fusion with the calmodulin-binding protein (CBP), we reported earlier (Oh et al., 2000) that CBP-BRI1 CD was unable to transphosphorylate kinase-inactive Flag-BRI1 (K911E). Moreover, the kinetics of autophosphorylation of CBP-BRI1-CD, measured as ^32^P-labeling from [^32^P]ATP, was first order with respect to [CBP-BRI1], providing two lines of evidence for an intramolecular reaction mechanism. In contrast, a subsequent study reported that GST-BRI1-CD could transphosphorylate the kinase-inactive BRI1 (K911E) mutant, suggesting an inter-molecular reaction (Wang et al., 2005b). The results of the present study suggest that BRI1 autophosphorylation may indeed involve both mechanisms, with autophosphorylation on some serine residues likely occurring as an intramolecular reaction while the majority of the serine, and all of the threonine and tyrosine sites, occurring via an inter-molecular reaction. This speculation is based on the observation that before induction with IPTG, bacterial cells produced some Flag-BRI1 protein but that protein had an overall low phosphorylation stoichiometry and autophosphorylation appeared to be restricted to serine residues (Figure 1). The increase in autophosphorylation following IPTG addition to the *E. coli* cells we suggest reflects the impact of increased concentration of recombinant protein in the bacterial cells and is consistent with autophosphorylation occurring via an inter-molecular reaction (Friedrichssen et al., 2000). Thus, the mechanism of autophosphorylation reported from *in vitro* experiments may be a function of the phosphorylation status of the recombinant protein at the beginning of the experiment and also the concentration of protein. A factor that may indirectly impact this latter aspect is the protein to which BRI1 is fused. In the experiments where intramolecular autophosphorylation was reported (Oh et al., 2000), BRI1 was fused to a monomeric protein, CBP, and the CBP-BRI1 protein used had a relatively low stoichiometry of autophosphorylation (based on identical SDS-PAGE mobility of active versus kinase-inactive protein). Both factors would be expected to promote primarily serine autophosphorylation via an intramolecular reaction, and indeed, both were reported (Oh et al., 2000). In contrast, expression of BRI1 fused to the dimeric protein, GST, might effectively increase the concentration of BRI1 protein and thereby promote inter-molecular autophosphorylation as reported earlier (Wang et al., 2005b). *In vivo*, BRI1 may exist to a certain extent as preformed homodimers in the absence of ligand (Hink et al., 2008), which would certainly promote autophosphorylation in particular via the inter-molecular reaction when BL is presented.

The transphosphorylation of *E. coli* proteins during expression of recombinant Flag-BRI1 (Figure 3) emerges as a potentially useful system to monitor the intrinsic kinase activity of BRI1 and other plant receptor kinases that display transphosphorylation activity. While a number of studies have reported autophosphorylation of recombinant protein kinases in *E. coli* (Yonemoto et al., 1993; Mattison et al., 2007), to our knowledge, none have reported transphosphorylation of *E. coli* proteins. From the preliminary results presented in Figure 5C it appears that some, but not all, receptor kinases may have this ability. Consequently, it will be of interest to further characterize this system. Also, recent studies with Flag-BRI1 and 14-3-3 proteins suggest that BRI1 preferentially transphosphorylates proteins to which it can bind (X. Wu, M.-H. Oh, S. D. Clouse and S. C. Huber, manuscript in preparation). Consequently, it would be of interest to determine whether this also applies to phosphorylation of bacterial proteins. It is worth noting that BRI1 tends to transphosphorylate *E. coli* proteins on threonine and tyrosine residues in particular that are in excess of ∼50-kDa; although there are numerous proteins with molecular weights below 50 kDa these are not transphosphorylated to nearly the same extent (Figure 5C). The basis for this apparent preference for larger proteins is not clear at present but will be investigated in the future. Finally, it also warrants comment that transphosphorylation of *E. coli* proteins tends to parallel the autophosphorylation of BRI1residues, with much greater phosphorylation on threonine relative to tyrosine residues (Figures 3B and **D**). This suggests that intrinsic tyrosine phosphorylation activity may be much more restricted or specific than serine/threonine phosphorylation; i.e., fewer tyrosine phosphorylation motifs are recognized. Alternatively, it may also reflect in part the fact that serine and threonine are together about fourfold more abundant in proteins compared to tyrosine. In the complete Expasy proteome database (www.expasy.org), the frequencies of serine, threonine, and tyrosine are 6.6, 5.3, and 2.9%, respectively. Insights learned from the bacterial system may help to answer these questions and thereby facilitate identification of novel plant proteins that are substrates of BRI1 *in vivo*.

The results reported in the present study have resolved the paradox of tyrosine autophosphorylation to the extent that the process has been observed to occur *in situ* (during expression of Flag-BRI1 in *E. coli*) and the mechanism established as a post-translational modification. Had the mechanism involved a co-translational modification, tyrosine phosphorylation would not have been a readily reversible modification and given that BRI1 is a relatively long-lived receptor kinase (Geldner et al., 2007), very different regulatory scenarios would need to be considered. However, explaining why tyrosine phosphorylation has not been observed *in vitro* in earlier experiments is not straightforward, but some explanations can be forwarded. In previous studies (Oh et al., 2000, 2009), the CBP-BRI1 protein used had a low level of autophosphorylation based on equivalent electrophoretic mobility relative to the kinase-inactive directed mutant CBP-BRI1 (K911E). Because of the hierarchical nature of autophosphorylation, ^32^P-labeling of tyrosine residues might have been restricted in these experiments. In the study by Friedrichssen et al. (2000), full-length BRI1-HA was transiently expressed in a mammalian cell line and immunoprecipitated BRI1 was used for the autophosphorylation studies. It is possible that the initial state of autophosphorylation and/or immobilization of the protein at the C-terminus interfered with tyrosine autophosphorylation. Finally, the study by Wang et al. (2005b) focused on autophosphorylation of the carboxy-terminus of BRI1 and perhaps because this region lacks tyrosine, only evidence for serine/threonine phosphorylation was reported. An overriding aspect that might play a role in all analyses is that, relative to serine/threonine, autophosphorylation on tyrosine is much more restricted in terms of number of sites and thus is inherently more difficult to detect.

Several other significant conclusions also emerged from this study relative to the role of the juxtamembrane domain. An overriding conclusion is that the juxtamembrane domain is indeed required for the majority of the autophosphorylation that occurs within the CD (Figure 7). Although not proven, it is likely that the juxtamembrane domain polypeptide interacts physically with the kinase domain, and that interaction is essential for kinase domain activity. Other molecular mechanisms are likely possible as well and will require additional experimentation to sort out. Regardless of the mechanism, it is clear that the juxtamembrane domain also affects specificity of BRI1 for autophosphorylation on serine/threonine versus tyrosine residues (Figure 7A). It is intrinsically easier to understand that residues within the kinase domain affect autophosphorylation specificity than it is to imagine that such effects can be exerted by flanking regions such as the juxtamembrane domain. However, both with truncations (Figure 7A) and targeted alanine scanning (Figure 8), we observed large effects of the juxtamembrane domain on autophosphorylation of BRI1 on tyrosine relative to threonine residues. Thus, one important and unexpected conclusion is that sequence motifs conferring tyrosine autophosphorylation (and thereby dual-specificity) might well be localized within the juxtamembrane domain as well as the kinase domain, where one would normally focus attention.

## Conclusion

Tyrosine autophosphorylation of recombinant Flag-BRI1 CD is shown to occur via a post-translational modification, as opposed to a co-translational modification, and the ability of BRI1 to transphosphorylate proteins on tyrosine residues (in addition to serine and threonine) is documented. It is interesting that transphosphorylation of *E. coli* proteins tends to parallel the autophosphorylation of BRI1 residues, with greater phosphorylation on serine/threonine relative to tyrosine. This suggests that tyrosine phosphorylation may be, in general, more restricted or specific than serine/threonine phosphorylation, which may also partially explain the inherent difficulty in monitoring tyrosine autophosphorylation. The phosphorylation of *E. coli* proteins provides a novel assay system to assess the intrinsic kinase activity of recombinant BRI1 CDs and will be useful in the future to characterize site-directed mutants. Finally, the juxtamembrane domain is confirmed as an activator of the BRI1 kinase domain and determinant of autophosphorylation specificity (serine/threonine relative to tyrosine).

## Conflict of Interest Statement

The authors declare that the research was conducted in the absence of any commercial or financial relationships that could be construed as a potential conflict of interest.

## Appendix

**Table A1:** **Identification of *E. coli* proteins phosphorylated on tyrosyl residues during expression of the recombinant Flag-BRI1 receptor kinase**.

	Protein	Expectation score	kDa	pI	Coverage (%)	Mr (expt)	Mr (calc)	Delta	Peptide sequence
Spot 1	Maltose transporter periplasmic protein	1.9 × 10^−4^	43.3	5.7	34	1188.682	1189.46	−0.778	AGLTFLVDLIK
						1336.582	1337.582	−1	VNYGVTVLPTFK
						1336.582	1336.421	0.161	SYEEELAKDPR
						1423.132	1423.586	−0.454	VTVEHPDKLEEK
						1766.112	1766.968	−0.856	FGGYAQSGLLAEITPDK
						1890.382	1891.281	−0.899	LIAYPIAVEALSLIYNK
						1913.392	1914.039	−0.647	HMNADTDYSIAEAAFNK
						2096.452	2097.306	−0.854	EFLENYLLTDEGLEAVNK
						2138.892	2139.439	−0.548	GQPSKPFVGVLSAGINAASPNK
Spot 2	Chain A of OmpFporin protein	4.0 × 10^−8^	37	4.7	43	719.222	718.894	0.328	LAFAGLK
						822.162	821.932	0.23	VGGVATYR
						1021.122	1021.184	−0.062	AVGLHYFSK
						1086.082	1086.169	−0.087	FTNTSGFANK
						1249.002	1249.302	−0.3	YADVGSFDYGR
						1370.042	1369.497	0.545	TNLQEAQPLGNGK
						1497.982	1497.672	0.31	TNLQEAQPLGNGKK
						1739.482	1739.943	−0.461	LGVGSDDTVAVGIVYQF
						1762.442	1762.791	−0.349	GNGENSYGGNGDMTYAR
						1847.902	1847.958	−0.056	YDANNIYLAANYGETR
						2133.382	2133.32	0.062	NMSTYVDYIINQIDSNK
						2149.322	2149.32	0.002	NMSTYVDYIINQIDSDNK
						2203.832	2203.482	0.35	NSNFFGLVDGLNFAVQYLGK

*Gel plugs analyzed are shown in Figure 4B*.

## References

[B1] Clouse S. D. (2011). Brassinosteroid signal transduction: from receptor kinase activation to transcriptional networks regulating plant development. Plant Cell 23, 1219–1230 10.1105/tpc.111.084475 21505068 PMC3101532

[B2] Cole A. Frame S. Cohen P. (2004). Further evidence that the tyrosine phosphorylation of glycogen synthase-3 (GSK3) in mammalian cells is an autophosphorylation event. Biochem. J. 377, 249–255 10.1042/BJ20031259 14570592 PMC1223856

[B3] Cowan S. W. Schirmer T. Rummel G. Steiert M. Ghosh R. Pauptit R. A. Jansonius J. N. Rosenbusch J. P. (1992). Crystal structures explain functional properties of two *E. coli* porins. Nature 358, 727–733 10.1038/358727a0 1380671

[B4] Cui J. Qasim S. Davidson A. L. (2010). Uncoupling substrate transport from ATP hydrolysis in the *Escherichia coli* maltose transporter. J. Biol. Chem. 285, 39986–39993 10.1074/jbc.M110.147819 20959448 PMC3000980

[B5] Ehsan H. Ray W. K. Phinney B. Wang X. Huber S. C. Clouse S. D. (2005). Interaction of *Arabidopsis* brassinosteroid-insensitive 1 receptor kinase with a homolog of mammalian TFG-β receptor interacting protein. Plant J. 43, 251–261 10.1111/j.1365-313X.2005.02448.x 15998311

[B6] Friedrichssen D. M. Joazeiro C. A. P. Li J. Hunter T. Chory J. (2000). Brassinosteroid-insensitive-1 is a ubiquitously expressed leucine-rich repeat receptor serine/threonine kinase. Plant Physiol. 123, 1247–1255 10.1104/pp.123.4.1247 10938344 PMC59084

[B7] Geldner N. Hyman D. L. Wang X. Schumacher K. Chory J. (2007). Endosomal signaling of plant steroid receptor kinase BRI1. Genes Dev. 21, 1598–1602 10.1101/gad.1561307 17578906 PMC1899468

[B8] Gendron J. M. Wang Z.-Y. (2007). Multiple mechanisms modulate brassinosteroid signaling. Curr. Opin. Plant Biol. 10, 436–441 10.1016/j.pbi.2007.08.015 17904409 PMC2093957

[B9] He J.-X. Gendron J. M. Sun Y. Gampala S. S. L. Gendron N. Sun C. Q. Wang Z.-Y. (2005). BZR1 is a transcriptional repressor with dual roles in brassinosteroid homeostasis and growth responses. Science 307, 1634–1638 10.1126/science.1107580 15681342 PMC2925132

[B10] Hink M. A. Shah K. Russinova E. De Vries S. C. Visser A. J. (2008). Fluorescence fluctuation analysis of *Arabidopsis thaliana* somatic embryogenesis receptor-like kinase and brassinosteroid insensitive 1 receptor oligomerization. Biophys. J. 94, 1052–1062 10.1529/biophysj.107.112003 17905839 PMC2186235

[B11] Jaillais Y. Hothorn M. Belkhadir Y. Dabi T. Nimchuk Z. L. Meyerowitz E. M. Chory J. (2011). Tyrosine phosphorylation controls brassinosteroid receptor activation by triggering membrane release of its kinase inhibitor. Genes Dev. 25, 232–237 10.1101/gad.2001911 21289069 PMC3034898

[B12] Johnson L. N. Noble M. E. Owen D. J. (1996). Active and inactive protein kinases: structural basis for regulation. Cell 85, 149–158 10.1016/S0092-8674(00)81092-2 8612268

[B13] Karlova R. Boeren S. Van Dongen W. Kwaaitaal M. Aker J. Vervoort J. De Vries S. C. (2009). Identification of in vitro phosphorylation sites in the *Aradopsis thaliana* somatic embryogenesis receptor-like kinases. Proteomics 9, 368–379 10.1002/pmic.200701059 19105183

[B14] Kim T.-W. Guan S. Burlingame Alma l. Wang Z.-Y. (2011). The CDG1 kinase mediates brassinosteroid signal transduction from BRI1 receptor kinase to BSU1 phosphatase and GSK3-like kinase BIN2. Mol. Cell 43, 561–571 10.1016/j.molcel.2011.07.031 21855796 PMC3206214

[B15] Kim T.-W. Guan S. Sun Y. Deng Z. Tang W. Shang J.-X. Sun Y. Burlingame A. L. Wang Z.-Y. (2009). Brassinosteroid signal transduction from cell-surface receptor kinases to nuclear transcription factors. Nature Cell Biol. 11, 1254–1262 10.1038/ncb1817 19734888 PMC2910619

[B16] Kjellström S. Jensen O. N. (2004). Phosphoric acid as a matrix additive for MALDI MS analysis of phosphopeptides and phosphoproteins. Anal. Chem. 76, 5109–5117 10.1021/ac0400257 15373450

[B17] Lochhead P. A. Kinstrie R. Sibbet G. Rawjee T. Morrice N. Cleghon V. (2006). A chaperone-dependent GSK3β transitional intermediate mediates activation-loop autophosphorylation. Mol. Cell 24, 627–633 10.1016/j.molcel.2006.10.009 17188038

[B18] Lochhead P. A. Sibbet G. Morrice N. Cleghon V. (2005). Activation-loop autophosphorylation is mediated by a novel transitional intermediate form of DYRKs. Cell 121, 925–936 10.1016/j.cell.2005.03.034 15960979

[B19] Mattison C. P. Old W. M. Steiner E. Huneycutt B. J. Resing K. A. Ahn N. G. Winey M. (2007). Mps1 activation loop autophosphorylation enhances kinase activity. J. Biol. Chem. 282, 30553–30561 10.1074/jbc.M707063200 17728254

[B20] Mora-Garcia S. Vert G. Yin Y. Cano-Delgado A. Cheong H. Chory J. (2004). Nuclear protein phosphatases with Kelch-repeat domains modulate the response to brassinosteroids in *Arabidopsis*. Genes Dev. 18, 448–460 10.1101/gad.1174204 14977918 PMC359398

[B21] Nam K. H. Li J. (2004). The *Arabidopsis* transthyretin-like protein is a potential substrate of brassinosteroid-insensitive 1. Plant Cell 16, 2406–2417 10.1105/tpc.104.023903 15319482 PMC520942

[B22] Nemhauser J. L. Hong F. Chory J. (2006). Different plant hormones regulate similar processes through largely nonoverlapping transcriptional responses. Cell 126, 467–475 10.1016/j.cell.2006.05.050 16901781

[B23] Oh M. H. Kim H. S. Wu X. Clouse S. D. Zielinski R. E. Huber S. C. (2012a). Calcium/calmodulin inhibition of the Arabidopsis BRI1 receptor kinase provides a possible link between calcium- and brassinosteroid-signaling. Biochem. J. 443, 515–523 10.1042/BJ20111871 22309147 PMC3316158

[B24] Oh M. H. Wang X. Clouse S. D. Huber S. C. (2012b). Deactivation of the *Arabidopsis* brassinosteroid insensitive 1 (BRI1) receptor kinase by autophosphorylation within the glycine-rich loop. Proc. Natl. Acad. Sci. U.S.A. 109, 327–332 10.1073/pnas.1108321109 22184234 PMC3252896

[B25] Oh M. H. Ray W. K. Huber S. C. Asara J. M. Gage D. A. Clouse S. D. (2000). Recombinant brassinosteroid insensitive 1 receptor-like kinase autophosphorylates on serine and threonine residues and phosphorylates a conserved peptide motif in vitro. Plant Physiol. 124, 751–766 10.1104/pp.124.2.751 11027724 PMC59180

[B26] Oh M. H. Wang X. Kota U. Goshe M. B. Clouse S. D. Huber S. C. (2009). Tyrosine phosphorylation of the BRI1 receptor kinase emerges as a component of brassinosteroid signaling in *Arabidopsis*. Proc. Natl. Acad. Sci. U.S.A. 106, 658–663 10.1073/pnas.0910766106 19124768 PMC2613937

[B27] Oh M.-H. Wang X. Wu X. Zhao Y. Clouse S. D. Huber S. C. (2010). Autophosphorylation of Tyr-610 in the receptor kinase BAK1 plays a role in brassinosteroid signaling and basal defense gene expression. Proc. Natl. Acad. Sci. U.S.A. 107, 17827–17832 10.1073/pnas.0915064107 20876109 PMC2955108

[B28] Tang W. Kim T. W. Oses-Prieto J. A. Sun Y. Deng Z. Zhu S. Wang R. Burlingame A. L. Wang Z. Y. (2008). BSKs mediate signal transduction from the receptor kinase BRI1 in Arabidopsis. Science 321, 557–560 10.1126/science.1156973 18653891 PMC2730546

[B29] Vert G. Nemhauser J. L. Geldner N. Hong F. Chory J. (2005). Molecular mechanisms of steroid hormone signaling in plants. Annu. Rev. Cell Dev. Biol. 21, 177–201 10.1146/annurev.cellbio.21.090704.151241 16212492

[B30] Wang X. Goshe M. B. Soderblom E. J. Phinney B. S. Kuchar J. A. Li J. Asami T. Yoshida S. Huber S. C. Clouse S. D. (2005a). Identification and functional analysis of *in vivo* phosphorylation sites of the *Arabidopsis* brassinosteroid-insensitive 1 receptor kinase. Plant Cell 17, 1685–1703 10.1105/tpc.105.031393 15894717 PMC1143070

[B31] Wang X. Li X. Meisenhelder J. Hunter T. Yoshida S. Asami T. Chory J. (2005b). Autoregulation and homodimerization are involved in the activation of the plant steroid receptor BRI1. Dev. Cell 8, 855–865 10.1016/j.devcel.2005.05.001 15935775

[B32] Wang Z. Y. Nakano T. Gendron J. He J. Chen M. Vafeados D. Yang Y. Fujioka S. Yoshida S. Asami T. Chory J. (2002). Nuclear-localized BZR1 mediates brassinosteroid-induced growth and feedback suppression of brassinosteroid biosynthesis. Dev. Cell 2, 505–513 10.1016/S1534-5807(02)00187-9 11970900

[B33] Wu G. Wang X. Li X. Kamiya Y. Otegui M. S. Chory J. (2011). Methylation of a phosphatase specifies dephosphorylation and degradation of activated brassinosteroid receptors. Sci. Signal. 4, ra29 10.1126/scisignal.2001258 21558554 PMC4854194

[B34] Yin Y. Vafeados D. Tao Y. Yoshida S. Asami T. Chory J. (2005). A new class of transcription factors mediates brassinosteroid-regulated gene expression in *Arabidopsis*. Cell 120, 249–259 10.1016/j.cell.2004.11.044 15680330

[B35] Yin Y. Wang Z. Y. Mora-Garcia S. Li J. Yoshida S. Asami T. Chory J. (2002). BES1 accumulates in the nucleus in response to brassinosteroids to regulate gene expression and promote stem elongation. Cell 109, 181–191 10.1016/S0092-8674(02)00721-3 12007405

[B36] Yonemoto W. Garrod S. M. Bell S. M. Taylor S. S. (1993). Identification of phosphorylation sites in the recombinant catalytic subunit of cAMP-dependent protein kinase. J. Biol. Chem. 268, 18626–18632 8395513

[B37] Zhang W. Chait B. T. (2000). ProFound: an expert system for protein identification using mass spectrometric peptide mapping information. Anal. Chem. 72, 2482–2489 10.1021/ac9911701 10857624

